# Native Perennial Grasses Show Evolutionary Response to *Bromus tectorum* (Cheatgrass) Invasion

**DOI:** 10.1371/journal.pone.0018145

**Published:** 2011-03-30

**Authors:** Erin M. Goergen, Elizabeth A. Leger, Erin K. Espeland

**Affiliations:** 1 Department of Natural Resources and Environmental Science, University of Nevada, Reno, Reno, Nevada, United States of America; 2 Northern Plains Agricultural Research Laboratory (NPARL), Agricultural Research Service (ARS) Sidney, United States Department of Agriculture (USDA), Montana, United States of America; Netherlands Institute of Ecology, Netherlands

## Abstract

Invasive species can change selective pressures on native plants by altering biotic and abiotic conditions in invaded habitats. Although invasions can lead to native species extirpation, they may also induce rapid evolutionary changes in remnant native plants. We investigated whether adult plants of five native perennial grasses exhibited trait shifts consistent with evolution in response to invasion by the introduced annual grass *Bromus tectorum* L. (cheatgrass), and asked how much variation there was among species and populations in the ability to grow successfully with the invader. Three hundred and twenty adult plants were collected from invaded and uninvaded communities from four locations near Reno, Nevada, USA. Each plant was divided in two and transplanted into the greenhouse. One clone was grown with *B. tectorum* while the other was grown alone, and we measured tolerance (ability to maintain size) and the ability to reduce size of *B. tectorum* for each plant. Plants from invaded populations consistently had earlier phenology than those from uninvaded populations, and in two out of four sites, invaded populations were more tolerant of *B. tectorum* competition than uninvaded populations. *Poa secunda* and one population of *E. multisetus* had the strongest suppressive effect on *B. tectorum*, and these two species were the only ones that flowered in competition with *B. tectorum.* Our study indicates that response to *B. tectorum* is a function of both location and species identity, with some, but not all, populations of native grasses showing trait shifts consistent with evolution in response to *B. tectorum* invasion within the Great Basin.

## Introduction

Invasion by non-native species poses a threat to native plant communities through mechanisms operating at multiple scales. At the landscape scale, non-native species can alter biogeochemical cycling, disturbance regimes, and trophic interactions [1]–[3]. At local scales, invasion by non-native species can affect the reproduction, abundance, or distribution of native species [4]-[5]. By altering the abiotic and biotic environment, invasive species are changing selection pressures experienced by native plants that remain in invaded communities [6]–[8]. Although altered conditions can lead to extirpation of some species, they can also induce rapid evolutionary changes in native populations that may increase the ability of natives to persist and coexist with non-native species [6], [8]–[13].

Restoration activities are often undertaken in an effort to reverse community changes in invaded systems, with mixed success [14]–[15]. Communities with a long history of disturbance may be among the most difficult and costly to restore due to changes in ecosystem properties and depletion of native species. However, isolated native plants often persist within disturbed environments, suggesting that these plants may possess traits that increase their performance in invaded conditions [6]–[7]. Comparing plants from invaded and uninvaded populations may provide insight on phenotypic traits that allow native plants to persist in invaded populations, and measuring changes in trait frequency can indicate whether invaded populations are potentially responding to selective pressures associated with invasion. While causal selective agents may be difficult to determine, a trait-based approach can allow us to identify phenotypes that will perform best when restoring invaded rangelands.

There is a growing consensus that native plant provenance should be considered when choosing genotypes to restore highly degraded communities [11], [16]–[17]. We believe that native plant performance, in addition to provenance, should also be considered. Specifically, two performance measures should be considered when selecting populations for restoring highly invaded systems. First, plants must be able to persist in invaded systems, tolerating shifts in biotic and abiotic conditions [15], [18]. Secondly, restored plants would ideally have a competitive effect on the invader (sensu Goldberg and Landa [19]), decreasing its abundance and potentially allowing restoration of other, less-tolerant native species. If native species can evolve increased tolerance and/or competitive effect in response to invasion, we may find valuable genotypes within remnant native populations [11]. However, not all species or populations will evolve in response to invasion. For adaptive evolution to occur, the invasive species must exert selection pressure on the native species, and the native species must possess heritable genetic variation upon which natural selection can act [6]. Additionally, factors such as the strength of selection, time since invasion, number of selection agents, population size, life history characteristics (e.g. mating system, lifespan, and time to reproduction), and gene flow are likely to affect evolutionary capacity of particular populations [6], [20]–[22].

In sagebrush steppe ecosystems of the Western USA, invasion by the exotic annual grass *Bromus tectorum* (cheatgrass) is leading to altered species composition, disturbance regimes, and soil biogeochemical cycling over an estimated 20,000 km^2^ of the Great Basin [1], [23]–[24]. Although other plant species have invaded the Great Basin, *B. tectorum* is the most dominant, covers the largest amount of land, and likely represents a strong and consistent selective pressure on native plants. *Bromus tectorum* germinates early, is highly competitive for limited water and nitrogen, and forms dense stands that limit re-establishment by native vegetation [25]. While seedlings of native species are typically not competitive with *B. tectorum,* established perennial species that are morphologically and phenologically similar to *B. tectorum,* like *Elymus elymoides, E. multisetus,* and *Poa secunda* (short-lived, early seral perennial grasses, [17], [25]–[26]) can limit *B. tectorum* establishment and reproduction [27]. Experimental evidence supports the importance of plant phenology in competition with *B. tectorum* [28]–[29], and evolutionary shifts towards earlier phenology have been observed in one population of *E. multisetus* [11]. Thus, these early-active native species may be better able to persist with *B. tectorum*.

In this experiment, we examined populations of five common native perennial grass species from four locations where paired invaded/uninvaded sites were found in close proximity, addressing the following questions: 1) Which species are the most tolerant of *B. tectorum* competition? 2) Which species exert the strongest competitive effect on *B. tectorum*? 3) Does native plant phenology differ between invaded and uninvaded populations? and 4) Are tolerant and/or competitive plants present in higher frequencies in invaded communities? Questions 1 and 2 address performance differences among species, while questions 3 and 4 allow us to infer whether trait shifts are consistent with an evolutionary response to invasion. By including multiple species and multiple locations, we were able to determine if certain species are consistently more tolerant of or competitive with *B. tectorum*, or if the capacity to evolve in response to invasion varies by location. Finally, we discuss the implications of an evolutionary response to invasion by native species for the conservation and restoration of invaded ecosystems.

## Methods

### Field and Greenhouse Methods

**Figure 1:**
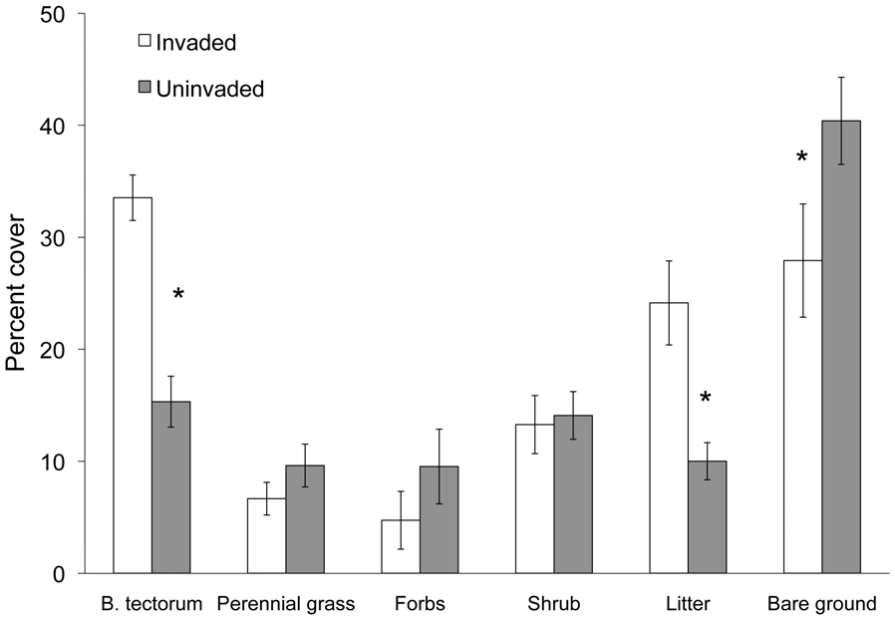
Community composition in invaded and uninvaded populations. Percent cover of *B. tectorum*, other plant functional groups, litter, and bare ground in invaded and uninvaded populations averaged over all four sampling locations. *Bromus tectorum* cover ranged from 2% in uninvaded areas to 40% in invaded areas. Asterisk indicates significant differences between invaded and uninvaded communities (*P*<0.05).

During December 4-11 2008, 40 adult plants of five perennial grass species, *Poa secunda*, *Elymus multisetus, Achanatherum hymenoides, Hesperostipa comata,* and *A. thurberianum,* were collected from four locations that had *B. tectorum* invaded and uninvaded areas in close proximity (Table 1). Potential sites within Carson City and southern Washoe County, Nevada, USA, were identified from University of Nevada herbarium collections that indicated *B. tectorum* presence for greater than 40 years. Twenty-five potential sites were visited. Many invaded areas had native plants growing with *B. tectorum*, but sites were only deemed suitable for this study if a native species was also present in an adjacent, uninvaded area with similar soils, slopes, and aspects. Four locations fit these criteria: Bedell Flats, Little Hill, McClellan Peak, and Tule Peak (Table 1, Fig. 1). At Little Hill and Tule Peak, a road separated invaded and uninvaded populations. In contrast, at Bedell Flats and McClellan Peak, there was no physical barrier separating the invaded and uninvaded populations but the boundaries between different communities were visually apparent and clearly distinct.

**Table 1:** Site and species characteristics of the four sampled locations.

	Bedell Flats	Little Hill	McClellan Peak	Tule Peak
**Lat Long**	39°49′58.10"N 119°45′56.10"W	39°52′51.20"N 119°42′55.60"W	39°14′21.30"N 119°44′34.70"W	39°54′0.10"N 119°42′4.90"W
**Elevation (m)**	1513	1335	1750	1470
**Mean Precip** + **(in)**	7.5	7.5	10.4	7.5
**Soil Type** †	Haybourne loamy sand	Washoe gravelly sandy loam	Indiano-Nosrac-Old Camp Association	Oppio cobbly sandy loam
**Parent Material** †	Alluvium derived from granitic rocks	Mixed alluvium	Residuum and colluvium derived from volcanic rock	Residuum derived from volcanic rock
**% Sand** †	73	67	47	35
**Size of uninvaded area relative to invaded area**	Small	Large	Equivalent	Large
**Species collected**	*Achnatherum hymenoides* 1,4 *Hesperostipa comata* 2,5 *Poa secunda* 1,3	*Achnatherum hymenoides*	*Achnatherum thurberianum* 2,4 *Elymus multisetus* 1,4 *Poa secunda*	*Elymus multisetus*

+ Precipitation data from Carson City Station (261485) for McClellan Peak and from Sutcliff Station (267953) for Bedell Flats, Little Hill, and Tule Peak (Western Regional Climate Center, www.wrcc.dri.edu).

† Soil data from Natural Resources Conservation Services, (websoilsurvey.nrcs.usda.gov).

1 Short-lived or ^2^long-lived species, and ^3^apomictic, ^4^selfing, or ^5^outcrossing mating system.

The invasion of *B. tectorum* in these four locations was likely a result of historic disturbance, which may have included fire, grazing, or physical disturbance. Thus, all potential selective agents were not manipulated individually. This is an experimental design constraint difficult to avoid in studies of historic invasion, particularly when disturbance is associated with invasion. Remnant native plants present in these disturbed sites may be survivors of the disturbance event, progeny of survivors, or represent re-colonization from surrounding communities. A 40+ year *B. tectorum* invasion likely represents more than one generation for the species we examined. Differences in trait means between invaded and uninvaded populations are interpreted as evidence consistent with evolution in response to invasion, and increased tolerance and/or competitive effect on *B. tectorum* in invaded areas supports the hypothesis that this evolution is adaptive. This experimental design cannot determine which event(s) were the strongest selective agents, nor the origin of tolerant/competitive genotypes, but can provide evidence that gene frequencies can shift in an adaptive manner in invaded populations.

Community composition at collection sites was recorded using a point-intercept method to measure percent cover of *B. tectorum*, dominant functional groups (perennial grasses, forbs, and shrubs), bare ground, and rock. Three parallel, 20-m transects, spaced 5 m apart, were located within the collection area, and point counts of all categories were recorded every 1 m. Soil characteristics for each site were identified (Table 1, http://websoilsurvey.nrcs.usda.gov) and verified by examining texture, percent coarse fragment, root biomass, and bulk density from three soil cores (0–90 cm) in both the invaded and uninvaded communities. Although soil characteristics differed among sites, there were no significant differences between invaded and uninvaded communities within sites (all *P*>0.14, data not shown).

Plant collections were determined by species occurrence in suitable sites. *Poa secunda*, *E. multisetus, and A. hymenoides* were collected from two locations, and *H. comata* and *A. thurberianum* were each sampled from one location (Table 1). To maximize the likelihood that differences between invaded and uninvaded populations within locations were due to *B. tectorum* invasion, plants were sampled from within a small area (∼0.5 km^2^). Using a shovel or a pickaxe, 20 individual plants per species were collected from both invaded and uninvaded areas with ∼ 2 L soil buffer around the crown. Plant phenology differed between collecting sites, and at the time of collection, most *P. secunda*, *H. comata*, *A. thurberianum*, and *E. multisetus* from Tule Peak had some green leaves. In contrast, *A. hymenoides* and *E. multisetus* from McClellan Peak were mostly dormant. The number of green leaves at collection was recorded. Forty plants per species were collected from each location, for a total of 320 plants.

Plants were transported to the University of Nevada, Reno greenhouses and transplanted into 1.65 L pots half-filled with a Nevada topsoil/sand/compost mix (Triple mix, RC Donovan, Reno, NV) within 24 h of collection. Each plant was divided into equal-sized halves (clones), placed in separate pots along with enough field soil (from the respective collection site) to fill the remainder of the pot, and watered. Each clone was randomly assigned to a control or competition treatment. Ten days after transplanting, ∼100 *B. tectorum* seeds were sown on the surface of each competition pot (density of ∼11,000 seeds m^−2^, comparable to the mid-range of field observations [26]). Pots were watered to saturation once per week and allowed to dry between watering, and plants were not fertilized. The experiment was concluded in mid-June 2009, when the majority of *B. tectorum* plants had set seed.

### Greenhouse Data Collection

The number of green leaves at planting (if present, Table 2) or, alternately, the date of leaf regrowth, and the date of initial inflorescence production (if produced) were recorded for each clone. At the conclusion of the experiment, the number of leaves and inflorescences produced were counted, and seeds/inflorescences were collected and weighed. To determine the competitive effect of each clone on *B. tectorum*, above-ground tissue of *B. tectorum* was collected from each pot, dried to a constant mass at 60°C, weighed to determine biomass and compared to average *B. tectorum* biomass without competition (measured in 19 pots where transplanted clones did not survive). We counted the number of *B. tectorum* seeds produced in a subset of 20 pots and used linear regression to estimate seed production, *y*, from dry biomass, *x*, using the following equation: 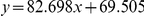, (*P*<0.0001, R^2^ = 0.89). The relative competitive performance index [30] was used to determine the tolerance of each native plant to *B. tectorum* competition:

**Table 2:** Results from ANOVA analysis showing the effect of collection location, species (nested in location), community type (invaded or uninvaded), and their interactions on response variables.

	Location		Species (location)		Community		Community x location		Species x community	
	*F*	*P*	*F*	*P*	*F*	*P*	*F*	*P*	*F*	*P*
a) % decline in leaves	2.67	**0.0481**	2.62	**0.0350**	2.64	0.1051	6.38	**0.0003**	1.03	0.3917
b) % decline in flowers*	11.61	**<0.0001**	3.98	**0.0485**	3.339	0.0679	2.50	0.0868	0.0069	0.9340
c) Bromus biomass	9.26	**<0.0001**	25.26	**<0.0001**	1.94	0.1651	11.05	**<0.0001**	5.37	**0.0003**
d) Initial # leaves‡	11.84	**<0.0001**	46.07	**<0.0001**	7.01	**0.0085**	1.45	0.2363	0.1653	0.9197
e) Days to green up†	42.07	**<0.0001**	32.83	**<0.0001**	7.92	**0.0052**	2.87	**0.0369**	2.58	0.0537
f) Days to flowering	21.06	**<0.0001**	57.3	**<0.0001**	2.06	0.1527	0.55	0.6477	5.64	**0.0002**

*P. secunda and E. multisetus only.

‡ Analysis includes plants green at initial collection, which were *P. secunda* (all at Bedell Flats and most at McClellan Peak), all *H. comata* (Bedell Flats), and most *A. thurberianum* (McClellan Peak) and *E. multisetus* (Tule Peak).

† Analysis includes only individual plants not green at initial collection, which were all *A. hymenoides* (Bedell Flats and Little Hill), most *E. multisetus* (McClellan Peak), and a few individuals of *P. secunda* (McClellan Peak), *A. thurberianum* (McClellan Peak) and *E. multisetus* (Tule Peak).



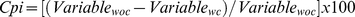
Leaf and inflorescence number were the response variables used, ‘woc’ is without competition, and ‘wc’ is with competition.

### Data Analyses

All analyses were conducted with JMP 5.0.1 (SAS Institute, Cary, North Carolina), and values presented are means ± standard error (SE). Differences in community composition among sampling locations and between community type (invaded or uninvaded) were compared using MANOVA, using the Wilk's lambda method of determining *F*, with location and community type as model effects and percent cover of vegetation as response variables. Green leaf number at collection or days to green-up, days to inflorescence production, leaf and inflorescence Cpi, and final *B. tectorum* biomass were analyzed using ANOVA with model effects of location, species (nested within location), community type, and their interactions. Only green leaf number at collection required log transformation to meet assumptions of ANOVA.

## Results

Community composition varied among individual sampling locations (*F*_21,38_ = 10.54, *P*<0.0001), and between community types (*F*_7,13_ = 21.53, *P*<0.0001). All invaded areas had greater cover of *B. tectorum*, greater amounts of litter, and less bare ground than uninvaded areas (Fig. 1). Shrub and forb cover was similar between invaded and uninvaded areas, and perennial grass cover was slightly, but not significantly, higher in uninvaded areas. Differences among sites were due primarily to variation in forb abundance: forbs were relatively absent at all locations except Bedell Flats, where annual forb cover averaged 20% in both community types.

**Figure 2:**
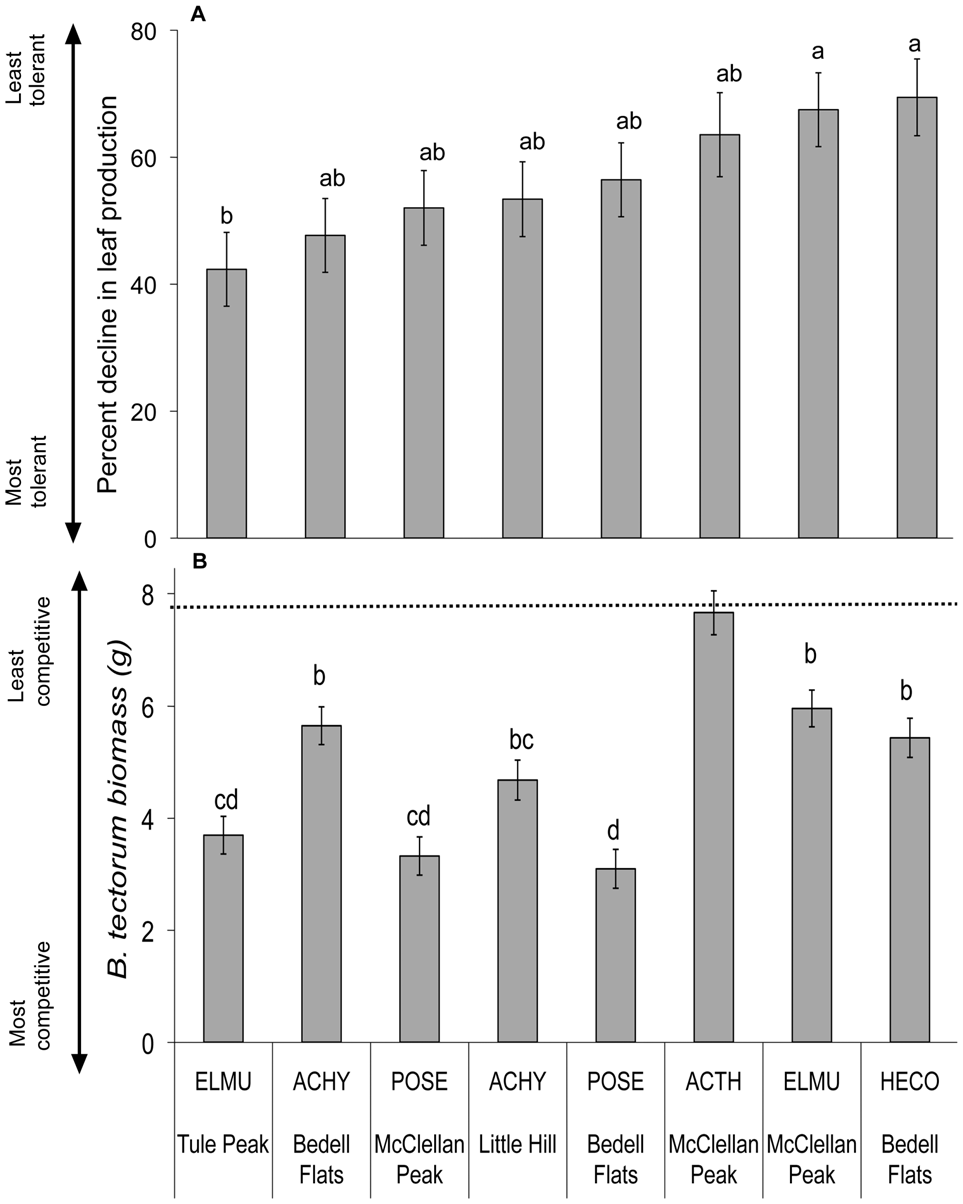
Tolerance and competitive effect of five native perennial grasses. Mean percent decline in biomass of target plants from Bedell Flats, Little Hill, McClellan Peak, and Tule Peak when grown in competition with *B. tectorum* (a) and biomass of *B. tectorum* when grown with target species (b). Dotted line indicates *B. tectorum* biomass when grown in monoculture (not included in analysis but plotted for comparison). Different letters within figures indicate significant differences (*P*<0.05) using Tukey adjusted least square means for multiple comparisons. POSE  =  *Poa secunda*, ACHY  =  *Achnatherum hymenoides*, HECO  =  *Hesperostipa comata*, ELMU  =  *Elymus multisetus*, and ACTH  =  *Achnatherum thurberianum.*

Native plants in competition with *B. tectorum* experienced an average 60% reduction in leaf number. However, the effect of *B. tectorum* competition on leaf production varied significantly by species and location (Table 2a, Fig. 2a). *Elymus multisetus* plants from Tule Peak were most tolerant of competition, averaging a 42% decline, whereas *E. multisetus* from McClellan Peak, along with *H. comata* from Bedell Flats, were among the least tolerant (67 and 69% declines, respectively). *Poa secunda* and *A. hymenoides* had intermediate tolerance (Fig. 2a).

Competition with *B. tectorum* reduced flower production. Although all species flowered when grown individually, only *P. secunda* and *E. multisetus* flowered when grown in competition with *B. tectorum*. The effect of *B*. *tectorum* on flowering also varied by location (Table 2b). *Elymus multisetus* from Tule Peak decreased inflorescence production by 48%, whereas plants from McClellan Peak had 94% reduction in flowering when grown in competition. *Poa secunda* from McClellan peak had 51% fewer inflorescences when grown with *B. tectorum*. In contrast, Bedell Flats *P. secunda* increased flower production by nearly 22% when grown in competition. Increased reproduction in *P. secunda* at this site was associated with a shift in biomass allocation: plants grown in competition with *B. tectorum* had decreased leaf to inflorescence ratio (greater than 50%) compared to plants grown without competition.

Perennial grasses differed in their ability to affect *B. tectorum* biomass (Table 2c, Fig. 2b), but tolerance and competitive effect were not tightly linked. Plants from populations that suppressed *B. tectorum* were not always the same populations that were most tolerant (Fig. 2a). Except for *A. thurberianum,* perennial grass presence reduced *B. tectorum* biomass by at least 20%. Tule Peak *E. multisetus* and both Bedell Flats and McClellan Peak *P. secunda* had the greatest competitive effect on *B. tectorum*, reducing its biomass by 53–60% (Fig. 2b). This competitive reduction in *B. tectorum* biomass resulted in 3–55% reduction in *B. tectorum* seed production.

**Figure 3:**
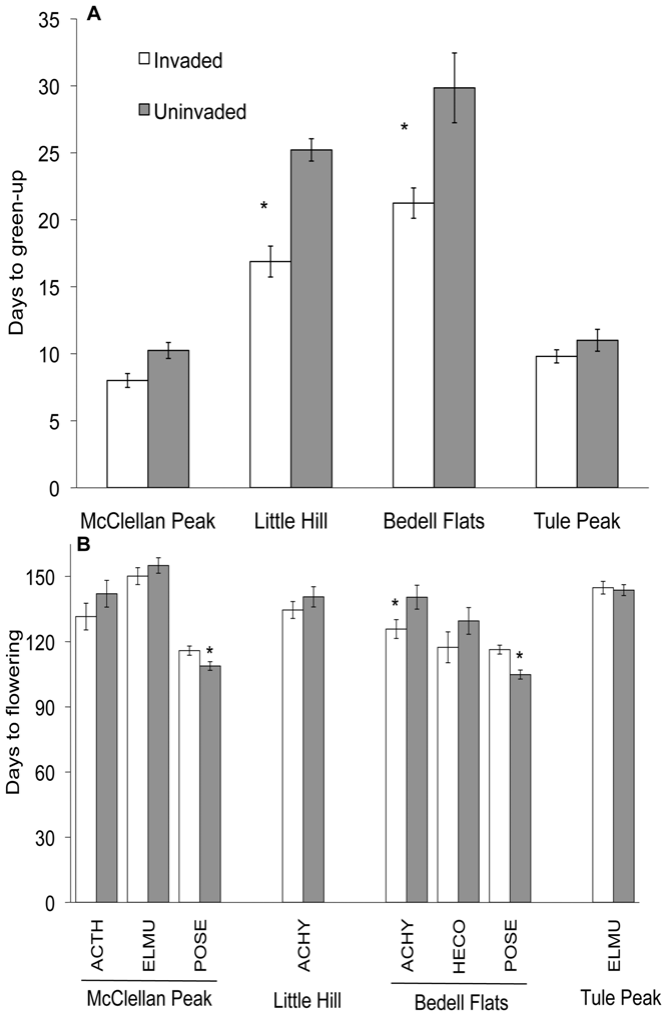
Phenology of invaded and uninvaded populations. Mean number of days before growth commenced for plants that were not green at the time of collection from invaded and uninvaded communities from Bedell Flats, Little Hill, McClellan Peak, and Tule Peak (a) and the number of days until flowering for each species from invaded and uninvaded communities at each sampling location (b). Asterisk indicates significant differences between invaded and uninvaded communities (*P*<0.05) based on post-hoc contrasts. POSE  =  *Poa secunda*, ACHY  =  *Achnatherum hymenoides*, HECO  =  *Hesperostipa comata*, ELMU  =  *Elymus multisetus*, and ACTH  =  *Achnatherum thurberianum.*

Native grasses collected from invaded populations had consistently earlier phenology. Actively growing plants collected from invaded communities had 18% more leaves at the time of collection than plants from paired uninvaded communities (Table 2d). The total number of leaves produced by the end of the season did not differ (invaded, 58.7±3.0; uninvaded, 52.4±3.1, respectively, *F* = 2.17, *P* = 0.174), indicating that growth likely commenced earlier in the season within invaded communities. Similarly, dormant individuals collected from invaded communities initiated growth in the greenhouse five days earlier than plants from uninvaded communities (Table 2e), significantly so in plants from Bedell Flats and Little Hill (Fig. 3a). Plants from invaded populations flowered qualitatively or significantly earlier than those from uninvaded populations (Table 2f). The exceptions were *Poa secunda* plants from uninvaded populations, which flowered on average five days before plants from invaded populations, and Tule Peak *E. multisetus* from both community types had similar flowering times (Fig. 3b).

**Figure 4:**
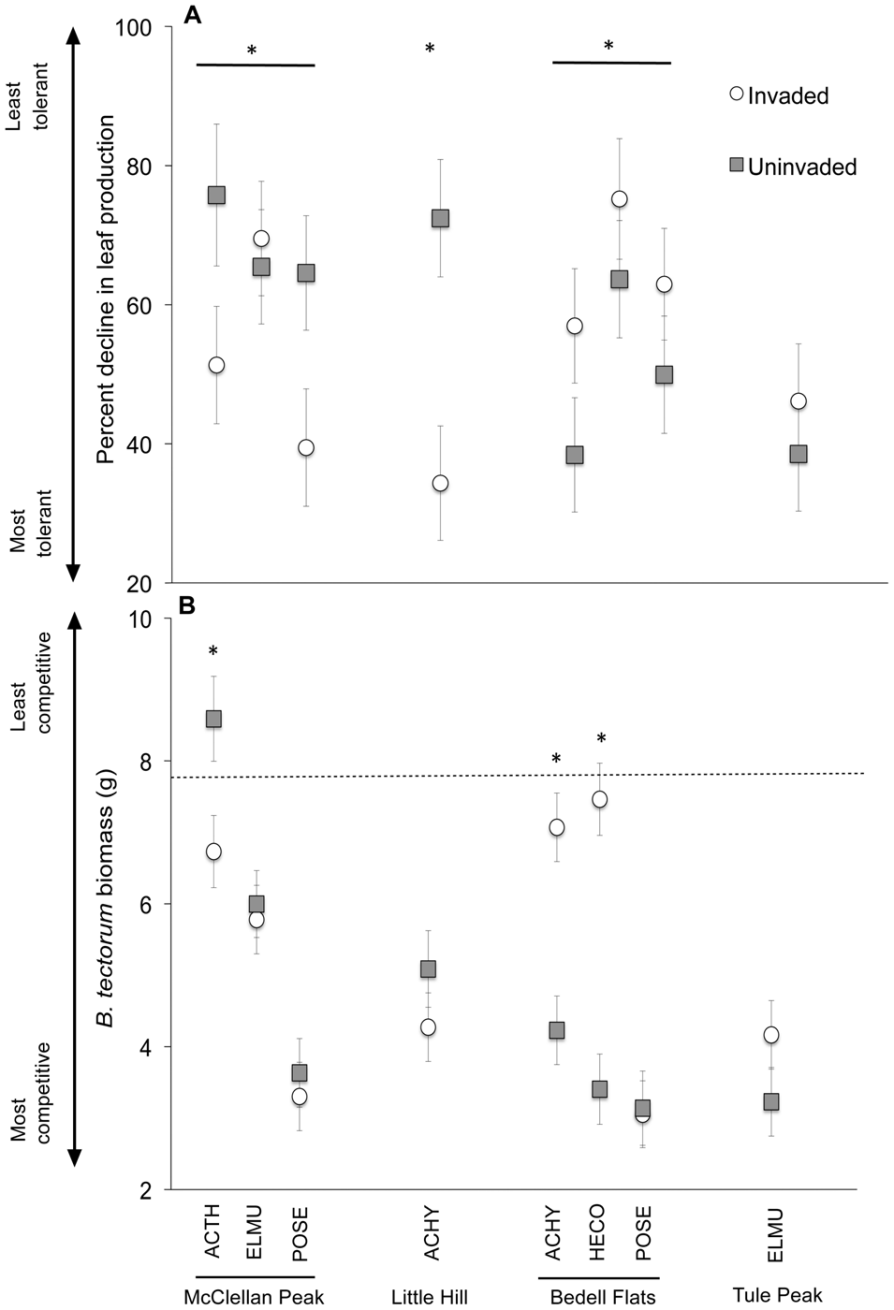
Tolerance and competitive effect of five native perennial grasses from invaded and uninvaded populations. Mean percent decline in biomass of target species from *B. tectorum* invaded and uninvaded communities at Bedell Flats, Little Hill, McClellan Peak, and Tule Peak when grown in competition with *B. tectorum* (a) and biomass of *B. tectorum* when grown with target species (b). Dotted line indicates *B. tectorum* biomass when grown in monoculture (not included in analysis but plotted for comparison). Asterisks indicate significant differences between invaded and uninvaded communities (*P*<0.05) based on post-hoc contrasts.

Plants from invaded communities did not consistently tolerate competition from *B. tectorum* better than plants from uninvaded communities (*P*>0.05, Fig. 4a), resulting in a community by location interaction (Table 2a). Plants from the invaded communities at McClellan Peak and Little Hill were significantly more tolerant of *B. tectorum* competition, producing 23–53% more biomass than plants from the uninvaded community. In contrast, plants from the uninvaded community at Bedell Flats showed greater tolerance, exhibiting significantly less reduction in leaf production (23%) when grown with *B. tectorum* compared to plants from the invaded community. There was no difference in leaf number Cpi between plants from invaded and uninvaded communities at Tule Peak, and *E. multisetus* from both communities were very tolerant to *B. tectorum* competition.

The competitive effect of perennial grasses also varied by collection site and community, but was not consistently greater in plants collected from invaded communities (Table 2c, Fig. 4b). Although *A. thurberianum* plants from the *B. tectorum* invaded community at McClellan Peak reduced *B. tectorum* biomass 22% more than plants from the uninvaded community, *A. hymenoides* and *H. comata* from the uninvaded community at Bedell flats reduced *B. tectorum* biomass 43–63% more than plants from the paired invaded community.

## Discussion

The widespread invasion of *B. tectorum* in the arid western US provides the opportunity to examine the response of native plants to the selective pressure of *B. tectorum* invasion in a variety of communities and across large areas. Results from our study indicate that some, but not all, populations of native grasses may be evolving in response to *B. tectorum* invasion within the Great Basin. Variation among populations in evolutionary response to invasion has been observed in other studies (e.g. [9]–[10]). Perennial grasses in two of our four study locations showed trait shifts in their ability to tolerate *B. tectorum* competition, consistent with a similar shift observed in *E. multisetus* from a nearby location [11]. Species examined in this study differed in their ability to suppress *B. tectorum,* but only in one collection did the plants from invaded areas have a significantly greater competitive effect than their uninvaded neighbors.

Native plants can persist in the face of invasion by *B. tectorum* by tolerating its presence (*i.e.* continue to perform relatively well in the presence of *B. tectorum*), through increased competitive suppression of *B. tectorum* (*i.e.* reduce performance of *B. tectorum*), or by a combination of both strategies. The strategy that leads to persistence may differ depending upon species traits, genetic diversity present within populations, and/or environmental site conditions [22]. In this study, we found that site, rather than species identity, was a larger factor in determining the tolerance of plants to *B. tectorum*. Although we expected *P. secunda* and *E. multisetus* plants to be the most tolerant of and competitive with *B. tectorum,* this was not consistent across locations and community types. Tolerance of *B. tectorum* presence and competitive effect on *B. tectorum* depended upon collection location for *E. multisetus* rather than community type (invaded versus uninvaded). *Poa secunda* was the most competitive species regardless of collection location or community type, yet its tolerance to *B. tectorum* varied by location and community type. Similarly, tolerance and competitive effect in *A. hymenoides* varied by location and community type. Site characteristics and site history are predicted to influence evolutionary capacity [6]–[7], [20]–[22], and the results of our study indicate that detailed studies of remnant native species occurring in sites with differing sizes, disturbance histories, and/or gene flow may be able to determine the field conditions that promote rapid evolutionary change.

In *B. tectorum* invaded communities, resources, especially water, are more available early in the season [31]. Thus, the ability to commence growth earlier in the season would likely increase resource capture and competitive ability of perennial species [32]–[33]. We found, for all species across all locations, plants either greened up earlier or had more green leaves at collection when collected from an invaded community, and most flowered earlier. However, the observed phenological shift was not always correlated with increased tolerance or competitive effect in the greenhouse (Table 2, Fig. 2). This may be due to the nature of a greenhouse experiment, where water is delivered in regular intervals and rooting depth is finite, which might preclude any adaptive value of early resource capture. In the field, where water resources peak in the spring and, via plant use, decrease over time, early phenology may indeed prove an effective way for perennials to usurp resources from annuals.

We expected plants from invaded areas to perform better in competition with *B. tectorum,* but the three species collected from the uninvaded community at Bedell Flats were significantly more tolerant of *B. tectorum* competition than plants from the invaded community. Bedell Flats had a large number of annual forbs present, even in uninvaded areas, and soils were sandier and had less coarse fragments and root biomass than the other sites. Additionally, unlike the linear border between invaded and uninvaded areas at other sites, at Bedell Flats the uninvaded area was surrounded by *B. tectorum*, and the relative size of the uninvaded area was much smaller compared to the other three sites. It is possible that *Bromus tectorum* was historically present within the “uninvaded” area and was being displaced by competitive plants, or, by chance, a patch of highly competitive plants precluded the colonization of *B. tectorum* in that small area.

Although the ability to tolerate competition and the ability to competitively suppress neighbors sometimes involves similar plant traits, these traits may not always overlap [19], [34]. For example, traits that confer tolerance to low resource availability (e.g. high resource use efficiency, low tissue turn-over) may not be the same traits that determine the ability of a plant to pre-empt resource capture (e.g. early phenology, high growth rates). We observed that, with the exception of *E. multisetus* from Tule Peak, the greatest tolerance and the highest competitive effect were not found within the same populations (Figure 2). This suggests that that using a mix of species or genotypes that includes both tolerant and competitive plants might result in the greatest restoration success in highly invaded environments.

In our experiment, as in other similar studies, it is not possible to completely rule out the potential role of non-genetic effects on phenotypic differentiation between plants. Non-genetic effects of parental plant environment on plant phenotypes (transgenerational plasticity, or maternal effects) are well documented, and include maternal seed provisioning or epigenetic influences on offspring phenotypes, resulting in non-heritable differences in plant phenotypes (reviewed in [35]). Plants growing in competition may have altered developmental pathways early in their development (e.g. [36]). If transgenerational plasticity or early developmental environment were at least partially responsible for the shifts in plant phenotypes observed across environmental gradients in this experiment, the influence of these factors varied among populations. This indicates that there is some genetic component to the patterns observed (e.g. [37], [38]). Mechanisms could be genetic variability in expression of transgenerational plasticity (e.g. [39]), genetic variability in phenotypically plastic response to the presence of a competitor (e.g. [40]), or genetic variability for specific non-plastic traits (local adaptation in the traditional sense, e.g. [41]). Whether competitive ability is determined more by environmental effects or genetic factors, the potential for invaded areas to be a source for restoration material remains a potentially powerful tool for more successful restoration in invaded areas.

This study and others indicate that native species may be responding to selection from the presence of invasive plants, and further, that there are both species- and population-level differences in the way native species perform with an invasive competitor [8]–[12]. In addition to examining the evolutionary effects of species invasion, these studies provide valuable information on potential restoration of invaded rangelands. In order to find the most tolerant and/or competitive genotypes in the Great Basin, we recommend wide-scale collections be undertaken from heavily invaded areas, as this and other studies have shown that these populations may be evolving in response to *B. tectorum*. Additionally, field performance in highly invaded areas should be a criterion for deciding which populations will be used for restoration. Finally, this study focused on adult traits, but restoration typically proceeds with seeding, rather than transplanting. In order for native plants to successfully survive in invaded communities in the long-term, populations must not only tolerate and compete with invaders as adults, but must also produce seeds that can establish in the invaded environment. Field studies are currently ongoing with the seed progeny of the plants examined in this study, and we will address whether seedlings of particularly tolerant/competitive individuals are also able to establish in competition with *B. tectorum*.
